# Germ Cell Proteins in Melanoma: Prognosis, Diagnosis, Treatment, and Theories on Expression

**DOI:** 10.1155/2012/621968

**Published:** 2012-11-12

**Authors:** Ashley M. Rosa, Nitika Dabas, Diana M. Byrnes, Mark S. Eller, James M. Grichnik

**Affiliations:** ^1^Department of Dermatology and Cutaneous Surgery, University of Miami Miller School of Medicine, Miami, FL 33136, USA; ^2^Anna Fund Melanoma Program, Sylvester Comprehensive Cancer Center, Miller School of Medicine, University of Miami, Room 912, BRB, 1501 NW 10th Avenue, Miami, FL 33136, USA; ^3^Department of Dermatology and Interdisciplinary Stem Cell Institute, Miller School of Medicine, University of Miami, Room 912, BRB, 1501 NW 10th Avenue, Miami, FL 33136, USA

## Abstract

Germ cell protein expression in melanoma has been shown to correlate with malignancy, severity of disease and to serve as an immunologic target for therapy. However, very little is known about the role that germ cell proteins play in cancer development. Unique germ cell pathways include those involved in immortalization, genetic evolution, and energy metabolism. There is an ever increasing recognition that within tumors there is a subpopulation of cells with stem-cell-like characteristics that play a role in driving tumorgenesis. Stem cell and germ cell biology is intertwined. Given the enormous potential and known expression of germ cell proteins in melanoma, it is possible that they represent a largely untapped resource that may play a fundamental role in tumor development and progression. The purpose of this paper is to provide an update on the current value of germ cell protein expression in melanoma diagnosis, prognosis, and therapy, as well as to review critical germ cell pathways and discuss the potential roles these pathways may play in malignant transformation.

## 1. Introduction

The primary objective of melanoma treatment is to specifically eradicate the tumor while minimizing damage to normal tissue. In order to accomplish this goal, it is necessary to identify tumor-specific targetable pathways. One group of proteins that exhibit selective expression in cancer includes a group of proteins whose expression is otherwise normally limited to germ cells. Most of the research into germ cell proteins in cancer has focused on expression differences and immunogenic potential for vaccines. However there is an increasing effort to decipher the potential role germ cell proteins may play in oncogenesis.

The first germ cell-specific antigen discovered was the Melanoma Antigen 1 (MAGE-A1) in a patient with prolonged survival despite bulky lymph node disease [1]. Ongoing research revealed a family of MAGE antigens expressed in many tumor types, and while also expressed in the testis, the antigens did not appear to be expressed in most normal adult tissues. With the expansion of known germ cell proteins in cancer, the term *cancer testis antigen *(CTA) was coined to refer to those proteins that are expressed primarily in the testis or placenta and cancers but not generally in normal adult tissues [2]. Nevertheless, CTA expression can be found in normal tissues, such as pancreas, brain, and liver [3]. The term CTA has been limited to proteins not expressed in more than two nongerm cell normal tissues [4]. However, clearly germ cell proteins may still play a role in cancer even if they are found to be more widely expressed.

There are now over 100 families and 255 cancer testis entries compiled in a database by Ludwig Institute for Cancer Research [5]. The common CTAs researched in cancer include MAGE, GAGE, and SSX families as well as NY-ESO1 and PRAME [3]. The large number of these proteins and their expression in cancer suggests a potential link between germ cell pathways and tumor development.

The developmental pathways leading to cancer are still being elucidated. However, it is now clear that melanoma tumors are heterogeneous, and subpopulations of cells with stem-cell-like features are present [6]. Germ cells may be considered the ultimate stem cells as they have the capacity to give rise to entirely new individuals. Some of the germ pathways are thought to have evolved from DNA repair pathways and serve to specifically create genetic diversity in offspring [7]. In single cell eukaryotes, such as yeast, these pathways are activated by stress [8, 9]. It is possible that germ cell pathways play a critical role in allowing tumors to become more genomically diverse, survive under different metabolic conditions, and extend overall growth potential.

Despite recent advances in melanoma care, patients continue to die from this disease. Germ cell proteins may hold the key to new therapeutic approaches to prevent the development and evolution of cancer. This review will focus on differences in germ cell protein expression, role in diagnosis, prognosis, and therapy. Further we will discuss critical germ cell pathways and consider the potential roles these pathways may play in malignant transformation.

## 2. Variable Expression of Germ Cell Proteins

Among malignancies, melanoma is one of the tumors with the highest frequency of CTA expression [3]. Also included in this group are bladder, lung, ovarian, and hepatocellular cancers. Expression is also found in basal cell and squamous cell carcinoma [10]. The lowest levels of expression appear to be in lymphomas, renal and colon cancers [3]. A correlation between the gene families expressed and cancer type has been identified. Melanoma has been noted to express higher levels of MAGEA, MAGEC3, SPANX, and LDH than other CTA gene families [3]. Further, expression of CTAs is often heterogeneous within a tumor [11]. The finding that germ cell proteins are not only differentially regulated across tumor types but they are also differentially regulated within tumor subpopulations suggests a potentially complex role in tumorigenesis.

## 3. Role in Diagnosis

Germ cell protein expression has a potential diagnostic role. This is critically important in melanoma as the diagnosis of early melanoma versus an unusual benign mole can be quite challenging. Melanoma diagnosis is dependent on dermatopathologist's visual review of tissue sections. A study by Farmer et al. on the discordance of the histopathologic diagnosis of melanoma found that 38% of the samples reviewed had two or more discordant diagnoses [12]. Recurrent nevi, combined nevi, acral nevi, deep penetrating nevi and Spitz nevi, may be overdiagnosed as melanoma while certain types of melanoma such as the nevoid, desmoplastic, Spitzoid, and regressed lesions may be underdiagnosed [13]. Misdiagnosis of certain lesions may lead to unnecessary invasive treatment or a malignant melanoma going untreated. In order to improve patient care it is vital to develop more accurate diagnostic tests that will aid dermatopathologists and clinicians in the difficult task of classifying ambiguous lesions.

The high expression of CTAs in melanoma and lack of expression in normal skin make the presence of these germ cell proteins a potential diagnostic tool (Table 1). Primary melanomas and benign nevi have been analyzed based on immune detection of three antigens: MAGE-A1 (MA454), MAGE-A4 (57B), and NY-ESO-1 (ES121). Approximately 50% of the melanoma samples tested positive for immunoreactivity with these 3 CTAs [14]. When the CTA panel was expanded to include three additional antigens including MAGE-C1 (CT7-33), MAGE-A3 (M3H67), and GAGE (GAGE), 77% of the melanomas tested positive for at least one CTA [14]. Therefore the sensitivity of potential diagnostic tests may be improved by increasing the number of antigens included in the array. Other studies have found 100% specificity for MAGE-3 in melanoma nodal metastasis under optimal PCR conditions, but determined the need for a broader panel of CTAs to increase the sensitivity [15]. One study that used only anti-MAGE antibody 57B found the diagnostic potential of CTAs was limited by their presence in several types of benign skin lesions [16]. This reinforces the need for a wider range of antibodies in order to improve diagnostic abilities.

Experiments have also been performed evaluating the germ cell protein SPANX. The expression pattern revealed a statistically significant difference between normal skin, benign nevi, and melanoma [17]. The prevalence of SPANX in 80.9% of the melanomas tested makes it a useful target for diagnostic assays. Normal skin did not show any presence of the antigen but benign nevi did display an intermediate level. This intermediate level was significantly lower than the expression in melanoma and significantly more than the expression in normal skin [17]. Additionally, MAGE-3 was found to be expressed in melanoma but not in normal melanocytes while PRAME was determined to be an adequate marker for differentiating between melanoma cells and benign nevi [15, 18]. The expression of PRAME in 88% of primary cutaneous melanomas and lack of expression in normal nevi make it particularly useful [19, 20].

Thus germ cell protein/CTA expression patterns may serve as a diagnostic tool to differentiate between malignant and benign skin lesions. 

## 4. Role in Prognosis

In addition to potential diagnostic value, germ cell proteins may also have prognostic value. After melanoma diagnosis, clinicians determine the relative prognosis in order to anticipate further care needs. Currently, prognostic information from the primary tumor is largely based on histopathologic criteria including Breslow's depth, ulceration, and mitotic rate. Nevertheless, some lesions that should have had a good prognosis by these variables ultimately prove to be lethal and some with a poor prognosis never recur. Thus there is need to better segregate the lethal and nonlethal lesions. A summary of CTAs and their associated prognostic features is presented (Table 2). According to the study by Svobodová et al. expression of CTAs has independent prognostic value for relapse-free survival (RFS), with CTA negative tumors (MAGE-A1, MAGE-A4, and NYESO-1) having an RFS of 72 months compared to 45 months for positive tumors [21]. This study found the CTA prognostic value comparable to the current clinic-pathologic classifications [21]. A study by Mikhaylova et al. revealed a correlation between the level of expression of CTAs and the differentiation of tumors with the more poorly differentiated cells exhibiting higher CTA levels [22]. 

Further a study found a specific prognostic relationship between the stage of melanoma and antigen present. According to Vourc'H-Jourdain et al., MAGE-A3 was significantly related to an increase in disease-free survival when expressed in stage III melanoma patients [23].

There is also potential prognostic value in determining the type of CTA present in the tumor. According to Velazquez et al. primary tumors that are NY-ESO1 positive are thicker than those tumors negative for this antigen [24], while Barrow et al. found NY-ESO1 expression in tumors of 1.1 to 4 mm significantly higher than in thin tumors less than 1 mm [25]. Also, the NY-ESO1 antigen is often more associated with metastatic disease [24, 26]. Combined thickness and metastasis properties result in a poor prognosis for NY-ESO1 positive tumors. When comparing the presence of NY-ESO1 to other antigens, like CTp11, there is a positive correlation between NY-ESO and more advanced disease while the CTp11 antigen is found in less advanced melanoma [26]. Certain antigens have been found exclusively in melanoma metastases and not in primary tumors, such as XAGE [27]. Another antigen, MAGE-A3, was expressed at least partially in 90% of metastases, making it a positive indicator for metastatic melanoma [28]. A study by Curioni-Fontecedro et al. found the expression of MAGE-C1 and MAGE-C2 in primary melanoma lesions to also be a significant predictor of lymph node metastasis [29]. Barrow et al. also found several correlations between MAGE-A1 and A4 and current prognostic criteria. MAGE-A1 showed a significant increase in expression in ulcerated and thicker tumors; although MAGE-A4 displayed a similar trend, its correlation was not statistically significant [25].

Thus CTA expression may have prognostic value in helping predict the potential aggressiveness of a tumor, potentially allowing clinicians to tailor therapeutic approaches accordingly.

## 5. Role in Treatment

Even without a complete understanding of germ cell protein function in cancers, there have been advances in germ cell protein-based therapies. However, until more is known about their function, the related therapies primarily revolve around germ cell protein immunogenicity. Tumor cells are generally considered antigenic but not immunogenic, allowing them to evade host immune defenses [30]. Recent cancer immunotherapy approaches focus on activating an immune response to cancer cell antigens. The limited expression of cancer testis antigens has made them a major target for immune-based therapies because their restricted expression should cause minimal side effects.

Melanoma-specific vaccines have been created to specific germ cell proteins in an effort to drive the patient's own immune system to attack the cancer cells. There are several current trials to test the efficacy of such vaccines in melanoma patients, particularly MAGE and NY-ESO1 antigens [31]. In addition to vaccines it may be possible to make the tumors more antigenic by driving the expression of CTAs. There is evidence that regulation of cancer testis genes, like MAGE, is dependent on DNA methylation and histone acetylation [32]. This makes epigenetic regulation by way of histone deacetylase inhibitors and DNA demethylating agents a possible treatment [32, 33]. KIT tyrosine kinase may also be important in the epigenetic control of MAGE and potentially a target for future therapies [34]. In addition, gene therapy could also be used. The study by Robbins et al. found T cell receptor modification of transduced T cells, specific for NY-ESO1, to be an effective therapeutic approach for melanoma [35]. Finally, combination therapies utilizing several of these approaches may have synergistic effect in treating melanoma.

In addition to the role of CTAs as direct immunologic targets, germ cell protein expression may also provide clinicians with insight into tumor sensitivity to certain drugs. Cell lines which express at least one of three MAGE genes are more susceptible to tumor necrosis factor-mediated cytotoxicity [36], while the over-expression of genes like MAGE-A2 and MAGE-A6 may signal resistance to chemotherapy [4]. The differences in susceptibility show the potential for tailoring therapies based on the expression of germ cell genes. 

Thus germ cell proteins may not only serve as direct immunologic targets but they may also direct the pharmacologic therapeutic approach.

## 6. Rationale for Germ Cell Protein Expression

While the presence of germ cell proteins in malignancies is well documented, the reason why they are expressed is not clearly understood. Theories on germ cell protein expression can be grouped into three general categories including (1) accidental/nonfunctional, (2) accidental/functional, and (3) programmed/functional.

The first is an accidental activation of germ cell-specific genes, which do not provide any functional benefit to the cancer cell. This scenario would include processes like widespread epigenetic regulation that would turn on germ cell genes secondarily but without any benefit to the cell. The most noted epigenetic controls in germ cell gene expression appear to be DNA methylation and posttranslational histone modifications [11].

The second category consists of the accidental activation of germ cell genes, but with a functional benefit to the cancer cells. By providing a benefit, the cancer cells that express these germ cell genes may survive and thrive better than those without expression thereby expanding the population expressing beneficial germ cell proteins.

Lastly, the expression of germ cell proteins may be programmed into the cell to be activated under certain conditions. These conditions could include hypoxia, lack of nutrients, and oxidative stress. Primitive organisms, like yeast, tend to increase genetic recombination when they experience such stresses in order to create new phenotypes that may be better suited for the environment [9]. A recent study by Forche et al. found a correlation between loss of heterozygosity in Candida Albicans and the degree of stress the yeast was exposed to, suggesting increased levels of gene rearrangement [8]. Due to overgrowth of the local blood supply, a fraction of cancer cells would be expected to be hypoxic and possibly nutrient deprived. An evolutionary conserved programmed response to the lack of oxygen and nutrients could lead to the activation of germ cell proteins, causing genetic recombination, and genomic instability, similar to that seen in yeast, allowing cancer cells to adapt to the environment and become more suited to thrive in adverse conditions.

In all three scenarios it would be expected that there would be differential expression of germ cell proteins in the tumor mass as the cancer progresses. Indeed, germ cell proteins are often heterogeneously expressed. Specifically, heterogeneity has been noted in the expression of SSX, GAGE, and NY-ESO1 [45–47]. This heterogeneity suggests the germ cell proteins are differentially regulated in the tumor cells. Evidence has been found for an increase in germ cell protein expression with tumor progression. A study by Barrow et al. found that both antigens MAGE-A1 and MAGE-A4 showed an increase in expression throughout disease progression [25]. Additionally, MAGE-A3 expression reveals a correlation with disease progression [37]. These patterns suggest that germ cell protein expression may be playing a role in tumor progression. The increase in expression may suggest that the germ cell phenotype confers adaptations more suitable for survival in adverse conditions. If these germ cell-specific genes are being turned on as a programmed response to stressors, their function is likely significant to survival of the cancer cells. Therefore, disrupting this function may decrease the ability of cancer cells to adapt and thrive under stressful conditions.

There is increasing evidence that germ cell pathways can be activated and may play a role in tumorigenesis. Hypoxic stress has been noted to induce germ cell protein expression in rat kidney fibroblasts, suggesting that hypoxia by itself may be enough to turn on some of these pathways [48]. The germ cell regulatory protein, PLU1 (JARID1B), has been shown to mark a subpopulation of melanoma tumor cells required for continuous tumor growth [49]. Further an association between germ cell gene expression and brain tumors in *Drosophila* has recently been identified [50]. Knock-down experiments of several of the germ cell proteins revealed that they played a critical role in tumor growth [50]. Together these studies support the idea that germ cell pathways may be activated due to stress or other means and that these proteins then functionally benefit the malignant state.

Although it is possible that germ cell protein expression is accidental/nonfunctional in cancer, given the association of upregulation with hypoxia and role in brain tumor development, it is more likely that expression of these pathways is programmed and functional in tumor development.

## 7. Cellular Pathways Affected by Germ Cell Proteins

Although there have been significant advances in unraveling the pathways involved in tumorigenesis, the role of germ cell proteins in this process remains to be fully understood. A recent review by Fratta et al. [11] covered many of the known molecular functions of CTAs; these findings will also be briefly addressed below in the apoptosis/transcriptional regulation sections. In addition to these findings, germ cell proteins affect other major potential pathways such as metabolism, meiosis, and telomere extension that are likely to play critical roles (Table 3). 

### 7.1. Prevention of Apoptosis

Genes of the class I MAGE family have been found to be associated with the p53 corepressor, Kap1. This complex between MAGE and Kap1 may suppress apoptosis in tumors [38]. The suppression of MAGE by siRNA and small compounds has been shown to inhibit tumor growth and induce apoptosis [51, 52]. The PRAME gene was found to repress retinoic acid signaling, a common proliferation inhibitor and apoptosis inducer [39]. By interfering with retinoic acid receptors, PRAME may upregulate proliferation and inhibit apoptosis. Thus expression of these germ cell proteins may help the cancer cells escape programmed cell death.

### 7.2. Transcription Control of Developmental Pathways Regulation

MAGE-A1 was found to inhibit transcription by interacting with the transcriptional regulator, SKIP, and recruiting histone deactlyase 1 (HDAC1) [40]. SKIP interacts with the NOTCH pathway, which controls cell differentiation during embryonic and adult life [11]. NOTCH signaling has been implicated in melanomagenesis [53]. Thus expression of germ cell proteins may promote tumor development.

### 7.3. Unique Energy Metabolism Pathways

Germ cells express a unique set of metabolic enzymes that allow them to utilize certain substrates more effectively. Spermatocytes are able to utilize lactate, pyruvate, and glucose while spermatids are only able to use lactate [54]. There are a number of glutamate transporters that are preferentially expressed in certain stages of spermatogenesis and allow for increased or decreased utilization of glucose. There are also specific glycolytic enzymes expressed only in spermatogenic cells, including hexokinase (*Hk*), phosphoglycerate kinase-2 (*Pgk2*), and glyceraldehyde 3-phosphate dehydrogenase (*Gapd*) [54]. Increased glucose utilization is an important finding in many cancers and has been referred to as the Warburg effect. In this phenomenon cancer cells utilize the glycolytic pathway in a fermentative manner, resulting in an increase in lactate [55]. Even in the presence of oxygen the cancer cells rely more on fermentation than respiration [55]. Diagnostic tools like the PET scan have exploited this trademark finding of cancer [56]. Expression of germ cell enzymes may contribute to the preferential use of glucose by cancer cells. 

The ability of germ cells to utilize lactate is also dependent on a testis-specific enzyme, Lactate Dehydrogenase C or LDHC [57]. Expression of this enzyme is found in numerous human cancers, allowing the cancers to use lactate as a substrate for ATP production [42]. This is beneficial to the cancer cells in light of the Warburg effect, which results in a buildup of lactate within the cell. Melanoma cell lines, when compared to normal melanocytes, rely more heavily on the Warburg effect [56]. While under hypoxic conditions melanocytes and melanoma cells both showed signs of glucose fermentation to lactate, only the melanoma cells were able to use the tricarboxylic acid cycle to produce fatty acids from glutamine and lactate from glucose [56]. The increase of lactate production and utilization in melanoma cells is accompanied by an upregulation of the germ cell protein LDHC. 

The change in metabolism seen in cancer cells is potentially attributable to this expression of alternate enzymes that are normally expressed in germ cells. Tumors may exploit these proteins to adapt to changes in substrate concentrations that accompany abnormal cell proliferation. This display of metabolic adaptations supports the theory of programmed expression of germ cell proteins because by expanding a cell's useable substrates for energy, the cell has an increased chance of survival.

### 7.4. Genetic Evolution/Meiosis/Aneuploidy

**Figure 1:**
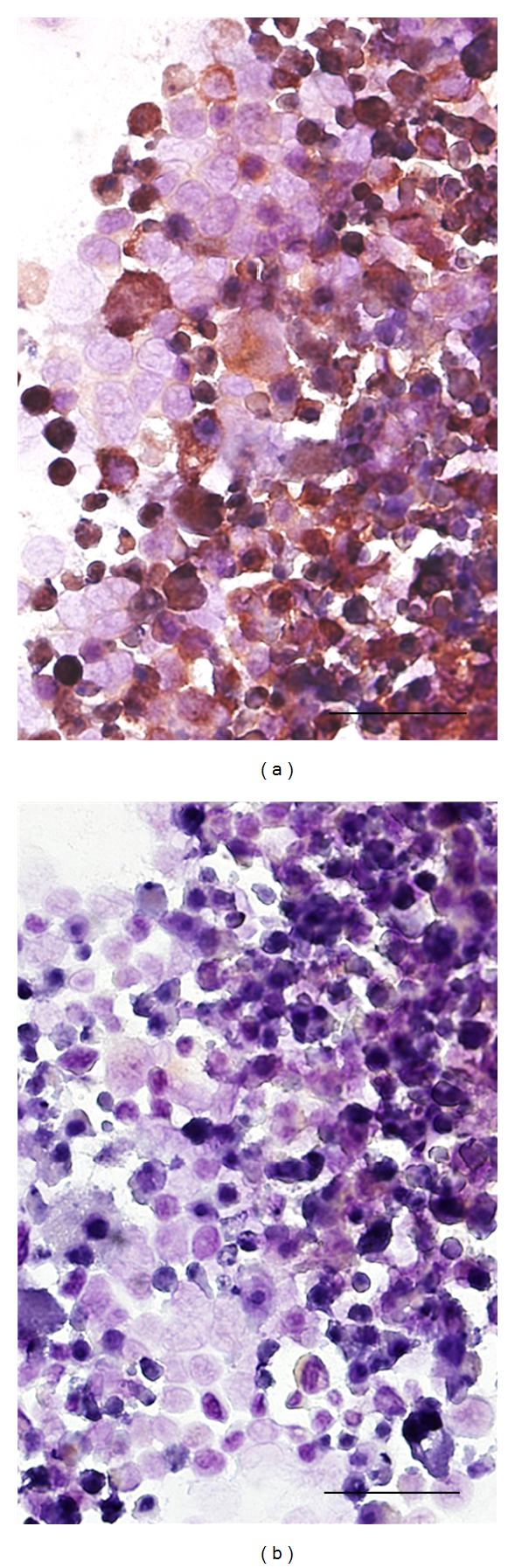
Germ cell proteins are often expressed in melanoma. Shown is a melanoma line DM2N stained with (a) and without (b) a primary antibody to REC8 (ProteinTech, Chicago, IL), a protein involved in chromosomal cohesion and crossover events in meiosis. The red staining (a) reveals that REC8 is abundantly expressed and is heterogeneous both in the level of expression and localization. It is possible that REC8 and other expressed germ cell proteins contribute to the chromosomal instability seen in melanoma and others tumors (size bar 50 *μ*m).

Cell division requires cell cycling through mitosis; however germ cells also have the capacity to undergo meiosis. Unlike mitosis, in the first meiotic division the sister chromatids remain attached to each other and recombination occurs across homologous chromosome arms. The expression of meiosis proteins in cells attempting to undergo mitosis (termed meiomitosis) could cause genomic instability [6]. Meiosis proteins have been documented to be expressed in melanoma. These include Spo11 a protein that creates double-strand DNA breaks [42]: SCP1, a protein involved in the pairing of homologous chromosomes [58] and HORMAD1 which may play a regulatory role in meiotic synapses [59]. There are also several other meiotic proteins that may be present in melanomas such as REC8, a meiosis-specific cohesion (Figure 1). During meiosis I REC8 is maintained at the centromeres and functions to bind the sister chromatids together [43]. In meiosis II REC8 is cleaved and the sister chromatids separate normally. The expression of REC8 during mitotic division could lead to failure of nuclear division, abnormal chromosomal segregation, and aneuploidy.

Thus meiosis proteins have been noted to be expressed in melanoma and could function to cause genomic instability.

### 7.5. Immortalization: TERT Expression

Since telomeres shorten with every round of division, cancer cells must express a mechanism to maintain telomere length in order to protect the genetic material [44]. In order to accomplish this task, over 90% of cancers reactivate telomerase, suggesting it is vital to their survival [44]. One of the highest rates of normal telomerase expression is in the testis just prior to meiosis I in the primary spermatocyte [60]. Thus telomerase is also an important germ cell protein. The levels of telomerase expression have been noted to be significantly higher in melanoma than in acquired and dysplastic nevi [61]. In contrast to cell lines that fail to continue to grow in culture, immortal melanoma lines maintain telomerase expression [62]. It is possible that the pathways leading to the activation of telomerase also activate other germ cell proteins.

In summary, the expression of germ cell proteins in melanoma has the capacity to prevent cell death, alter transcription, improve energy options, promote evolution of the genome, and provide infinite growth potential.

## 8. Discussion

Germ cell proteins are expressed in melanoma and numerous other malignancies. The expression of these proteins in melanoma has been demonstrated to have both diagnostic and prognostic value. Therapeutically research is advancing based on CTA vaccines. However, the role of germ cell proteins during oncogenesis is still largely unknown. It is likely that they contribute to altered metabolism, immortalization, and genomic instability in melanoma and other cancers. The high level of expression of germ cell proteins within melanoma makes the disease an ideal model for dissecting their role in tumorgenesis. These efforts may provide insights into microenvironmental processes that differentially regulate the germ cell genes which in turn likely serve to drive tumorgenesis. Ultimately research unraveling these pathways may allow for the development of new therapeutic targets to control or potentially eradicate tumor cell growth.

## Figures and Tables

**Table 1:** CTA diagnostic potential.

	% MM positive	% BN positive	% NS positive	Reference
3 CTAs	50	0		[14]
6 CTAs	77	0		[14]
SPANX	80.9	25	0	[17]
PRAME	88		0	[19, 20]
MAGE*		49		[16]

MM: melanoma; BN: benign nevi; NS: normal skin % of samples positive for expression of gene.

3 CTAs tested MAGE-A1, MAGE-A4, and NY-ESO-1.

6 CTAs tested MAGE-A1, MAGE-A4, NY-ESO-1, MAGE-C1, MAGE-A3, and GAGE.

*Anti-MAGE antibody 57B.

**Table 2:** Cancer testis gene and associated melanoma prognostic marker.

CTA	Thickness	Ulceration	Metastasis	RFS	Reference
NY-ESO1	***√***		***√***	−	[21, 24, 25]
MAGE-A1	***√***	***√***		−	[21]
MAGE-A4				−	[21]
XAGE-1			***√***		[27]
MAGE-A3			***√***	+*	[23, 37]
MAGE-C1/C2			***√***		[29]

***√***: the gene is associated with thicker tumors, ulcerated tumors, and metastatic tumors.

(−) A decrease in time of relapse free survival.

(+) increase in time of relapse free survival.

*Only found in stage III melanoma.

**Table 3:** Germ cell proteins expressed in cancer and proposed function.

Gene	Germ cell function	Cancer function	Mechanism	Reference
Class I MAGE	Apoptosis inhibition	Proliferation	Kap1-p53 corepressor	[38]
PRAME	Apoptosis inhibition	Proliferation	Retinoic acid receptor	[39]
MAGE-A1	Transcriptional regulation	Aberrant transcriptional regulation	SKIP, HDAC1	[40]
GAGE	Gene expression regulation	Aberrant gene expression		[41]
LDHC	Lactose metabolism	Metabolic efficiency		[42]
SPO-11	Recombination	Chromosomal instability	Double-stranded breaks	[4]
SCP-1	Recombination	Chromosomal instability	Homologous pairing	[4]
REC8*	Chromosome segregation	Aneuploidy	Regulated cohesion expression	[43]
TERT*	Genome protection	Immortalization	Telomere lengthening	[44]

*Germ cell proteins also expressed in cancer but not currently defined as CTAs.
